# Application of bacteriophage as food preservative to control enteropathogenic *Escherichia coli* (EPEC)

**DOI:** 10.1186/s13104-021-05756-9

**Published:** 2021-08-28

**Authors:** Diana Elizabeth Waturangi, Cecillia Pingkan Kasriady, Geofany Guntama, Amelinda Minerva Sahulata, Diana Lestari, Stella Magdalena

**Affiliations:** 1 Department of Biotechnology, Atma Jaya Catholic University of Indonesia, Jalan Jenderal Sudirman 12930, Jakarta, Indonesia; 2 Department of Food Technology, Atma Jaya Catholic University of Indonesia, Jalan Jenderal Sudirman 12930, Jakarta, Indonesia

**Keywords:** Bacteriophages, Enteropathogenic *Escherichia coli* (EPEC), Foodborne disease, Food preservative

## Abstract

**Objectives:**

This study was conducted to characterize lytic bacteriophages infecting enteropathogenic *Escherichia coli* (EPEC) on several types of food and analyze their ability as phage biocontrol to be used as a food preservative. Characterization was done for bacteriophage morphology and stability, along with the determination of minimum multiplicity of infection (miMOI), and application of bacteriophage in the food matrix.

**Results:**

Out of the five samples, BL EPEC bacteriophage exhibited the highest titer of 2.05  ×  10^9^ PFU/mL, with a wide range of pH tolerance, and high thermal tolerance. BL EPEC also showed the least reduction after 168 h of incubation, with a rate of 0.90  ×  10^–3^ log_10_ per hour. Bacteriophages from BL EPEC and CS EPEC showed an ideal value of miMOI of 0.01. As a food preservative, BL EPEC bacteriophage was able to reduce bacteria in food samples with a reduction above 0.24 log_10_ in lettuce and approximately 1.84 log_10_ in milk. From this study we found that BL EPEC bacteriophage showed the greatest potential to be used as phage biocontrol to improve food safety

## Introduction

Pathogenic bacteria such as Enteropathogenic *Escherichia coli* (EPEC) are one of the main cause of foodborne disease, and it also has been reported as the major source of many foodborne disease cases. In 2010, it was estimated that foodborne disease had caused the death of more than 4,20,000 people globally [1, 2]. EPEC can infect and transmit into the human body through contaminated food due to the poor sanitation, therefore it can bring a big impact on public health, especially outbreak diseases [3]. Many strategies have been used to reduce the contamination of pathogenic bacteria in food, such as the use of various chemical preservatives, but some of them came with unwanted side effects. Therefore, an alternative solution offered is to use bacteriophage to control food borne pathogens for food preservatives to improve food safety [4].

Bacteriophages have the potency to be used as a natural food preservative mainly due to their high specificity, self-replicating, and rapid killing property towards the pathogenic bacteria [5]. In applying bacteriophages in various forms of food, it should have characteristics that are suitable to be used as a food preservative, such as lytic activity towards pathogenic bacteria, not altering the quality of the food [6, 7] and have a broad spectrum to cover all important target bacteria [8]. The objectives of this research were to characterize lytic bacteriophages infecting EPEC, morphology determination, stability and to analyze their effectiveness on several types of food.

## Main text

### Methods

#### Bacterial refreshment

Enteropathogenic *Escherichia coli* Nmr-2 from Namru-2 was used in this research as the bacterial host. EPEC Nmr-2 was inoculated onto Luria Bertani (LB) agar and incubated at 37 °C overnight.

#### Bacteriophage enrichment and purification

EPEC Nmr-2 was inoculated into LB broth and incubated for 6–8 h to obtain the mid-log growth phase [9]. We used 5 bacteriophage of EPEC which were isolated from previous studies including (K EPEC) from ketoprak, (BI EPEC) from beef intestine (BL EPEC) from beef lung, (CI EPEC) from chicken intestine and (CS EPEC) from chicken skin. Bacteriophage was enriched by adding previously grown bacteria and specific bacteriophage into LB broth and incubated overnight [10]. After enrichment, the mixture was centrifuged at 10,000×*g* for 10 min. The supernatant was filtered using a 0.22 μm pore-size membrane syringe filter [9].

#### Bacteriophage titer determination

Titer was determined using the double-agar overlay method. Bacteriophage lysate was diluted using SM buffer with a series of tenfold dilution. Visible plaque was calculated and converted into plaque forming unit per milliliter (PFU/mL) [9].

#### pH and thermal stability assay

For pH stability assay, bacteriophage lysate was added into a series of tubes containing SM buffers (pH 2, 4, 6, 8, 10, 12, and 14). As for thermal stability assay, bacteriophage lysate was incubated at different temperatures (4, 25, 37, 45, 55, 60, 65, and 70 °C). Titer was determined using the double-layer agar method [11].

#### Morphology analysis

Bacteriophage morphology was determined using the Transmission Electron Microscope (TEM) at Eijkman Institute of Molecular Biology, Jakarta. Bacteriophage lysate was dropped onto a 400-mesh grid and was negatively stained using 2% (w/v) uranyl acetate on carbon-coated grids. Grids were observed using JEM-1010 TEM (JEOL, Tokyo, Japan) at magnification of  ×  30,000 [10].

#### Storage stability analysis

Bacteriophage stability was determined by incubating bacteriophage lysates at 120 rpm, 37 °C and were taken in 0, 1, 5, and 7 days. Titer was determined using the double-layer agar method [11]. Bacteriophage reduction rate was calculated with the formula below:

Reduction rate  = $$\frac{T- {T}_{0}}{t}$$

T  =  Bacteriophage titer after 168 h of incubation.

T_0_  =  Bacteriophage initial titer.

t  =  Time of incubation.

#### Determination of minimum multiplicity of infection (miMOI)

EPEC Nmr-2 was grown in LB broth overnight. Absorbance of bacteria was measured at OD_600_  =  0.132 (~ 10^8^ CFU/mL) and diluted to 10^6^ CFU/mL. Bacteriophage sample was diluted to 10^3^ PFU/mL. Bacteria and bacteriophage lysates were added into microplate wells with MOI ratio from 0.001 to 1000 and were done in duplicate. Absorbance was measured using a microplate reader for 12 h [12].

#### Application of bacteriophage

Shrimp, chicken meat, milk, and tofu were sterilized using an autoclave, whereas lettuce was sprayed with 70% alcohol. EPEC Nmr-2 was grown in LB broth and diluted to 10^6^ CFU/m. Bacteria and bacteriophage lysate (MOI 0.01) were added to the samples and incubated at room temperature and 4 °C, overnight. Lysates were diluted in SM buffer and enumerated using spread plate method to count the bacterial reduction [5, 13].

## Results

### Bacteriophage titer

A total of 5 bacteriophages were successfully purified which were K EPEC, BI EPEC, BL EPEC, CI EPEC and CS EPEC. From this study, we found that BL EPEC bacteriophage showed the highest titer with a titer value of 2.05  ×  10^9^ PFU/mL. The lowest titer was shown by BI EPEC bacteriophage with a titer of 3.33  ×  10^8^ PFU/mL. Whereas K EPEC was 1.57  ×  10^9^ PFU/mL, CI EPEC was 1.40  ×  10^9^, and CS EPEC was 1.67  ×  10^9^ PFU/mL.

### pH and thermal stability assay

**Fig. 1:**
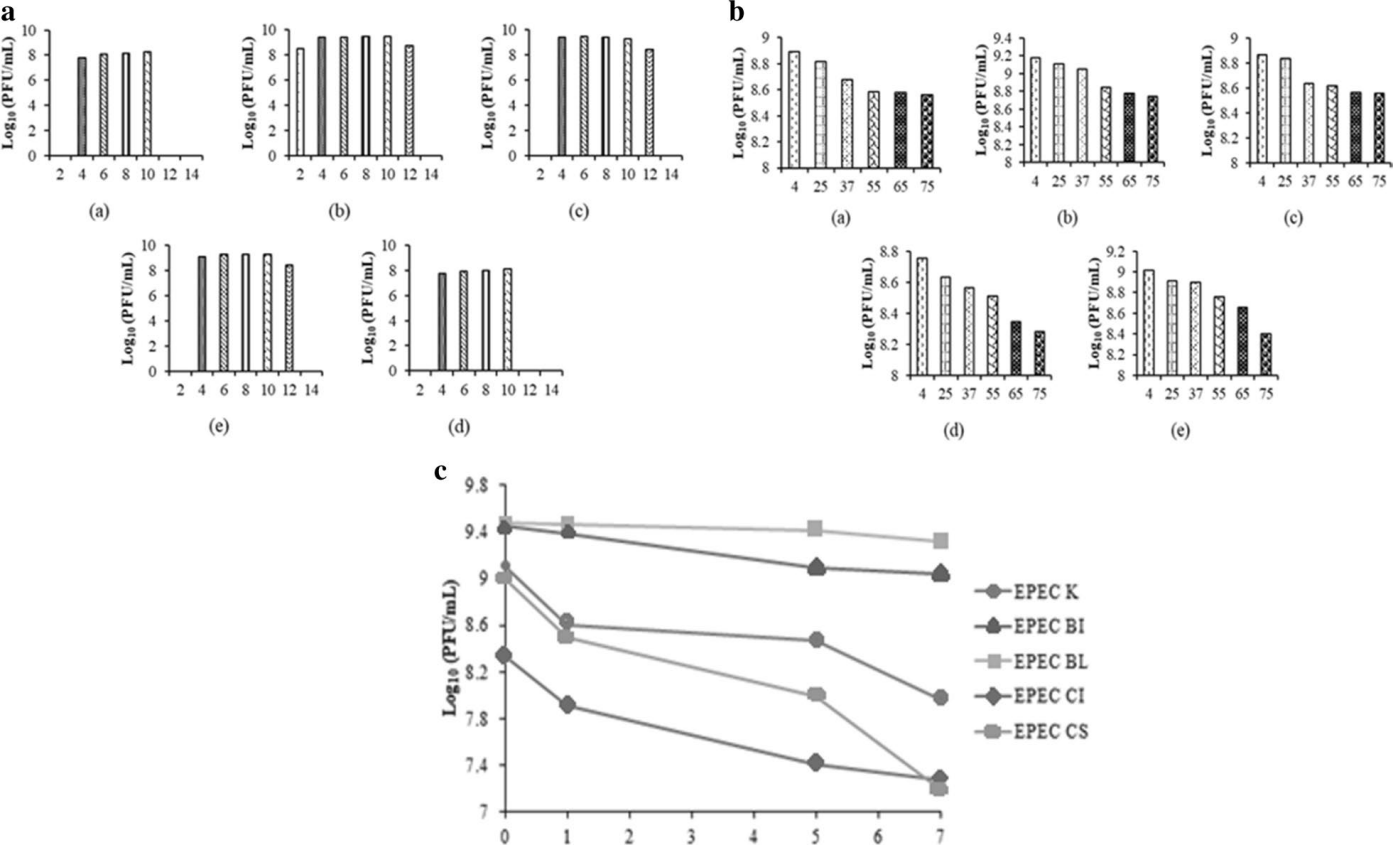
Stability of bacteriophage (a) K EPEC, (b) BI EPEC, (c) BL EPEC, (d) CI EPEC, (e) CS EPEC. **a** Stability on different pH values **b** Stability on different temperatures **c** Stability of bacteriophage during storage without the presence of its bacterial cell host

All of bacteriophage were stable upon exposure to pH 4–10, whereas there were no recoverable bacteriophage upon exposure to pH 14 (Fig. 1a). All of Bacteriophage were also stable upon heating from 4 to 75 °C with the highest titer found from treatment at 4 °C (Fig. 1b). Bacteriophage titer was constantly declining with the rise of temperature.

### Morphology analysis

**Fig. 2:**
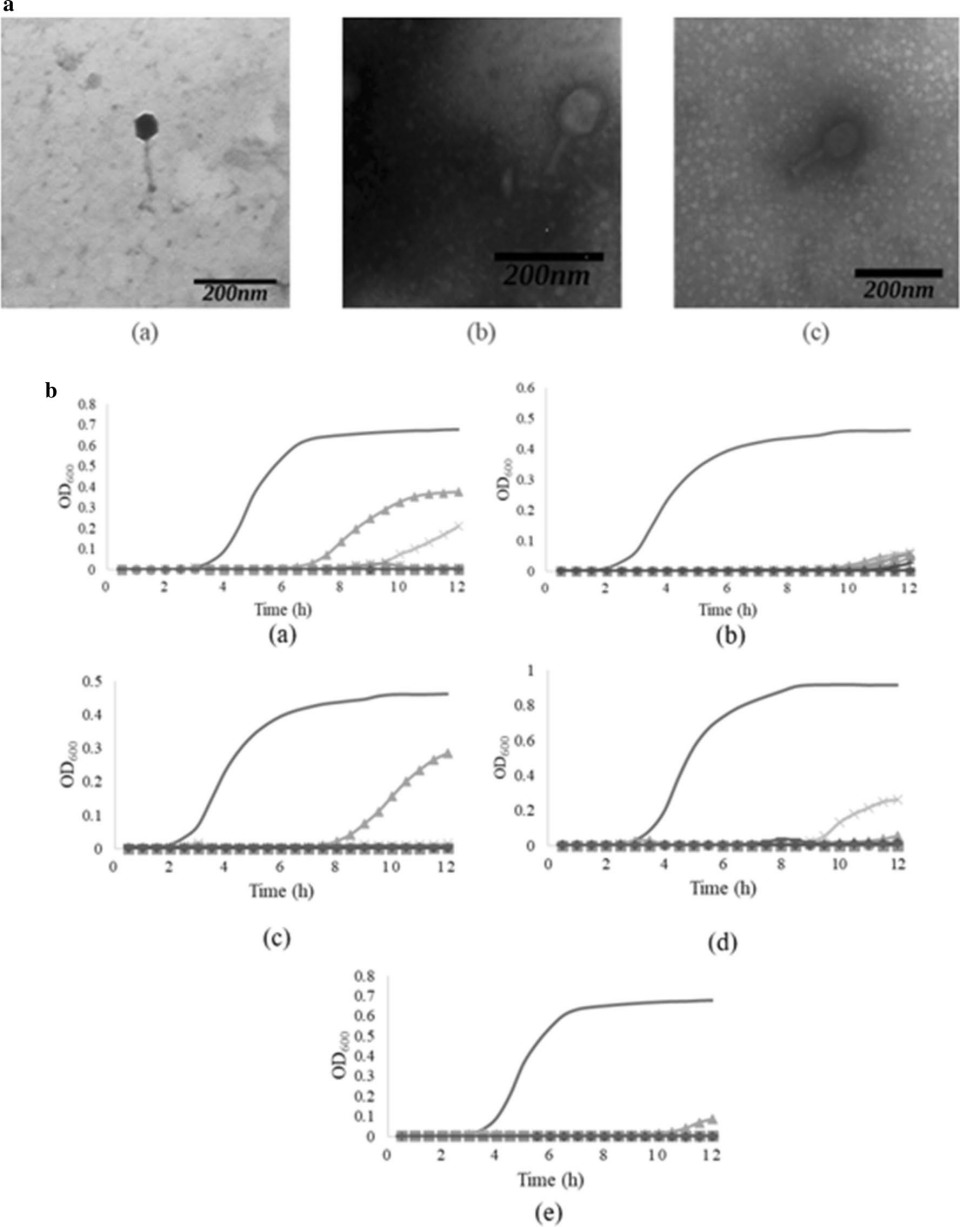
**a** Electron micrographs of bacteriophages (a) K EPEC, (b) BI EPEC, (c) CS EPEC. **b** The effect of several MOI ratio of bacteriophages (a) K EPEC, (b) BI EPEC, (c) BL EPEC, (d) CS EPEC, and (e) CI EPEC against EPEC bacteria’s growth

Morphology analysis was done for K EPEC, BI EPEC, and CS EPEC bacteriophage, since the analysis using TEM for BL EPEC and CI EPEC bacteriophage had been done in previous study [10]. TEM results showed that all bacteriophage samples had an icosahedral head and a tail (Fig. 2a). Based on the measurement, the tail diameter for K EPEC bacteriophage was 10 nm, 19 nm for BI EPEC, and 17 nm for CS EPEC, while the tail length was 133 nm for K EPEC, 82 nm for BI EPEC, and 86 nm for CS EPEC.

### Storage stability analysis

Deterioration of viable bacteriophage titer was occurred after 168 h of incubation without the presence of a bacterial host. BL EPEC bacteriophage showed the least reduction with a reduction of 0.16 log10 and reduction rate of 0.90 × 10^–3^ log_10_ per hour while CS EPEC bacteriophage showed the most reduction with a reduction of 1.80 log_10_ and reduction rate of 1.08  ×  10^–2^ log_10_ per hour (Fig. 1c).

### Minimum inhibitory multiplication of infection (miMOI)

The greatest MOI tested was 1000 which was equivalent to 1000 PFU per 1 CFU and the least MOI tested was 0.001. BL EPEC and CS EPEC had the least miMOI, which were 0.01, BI EPEC was 10 and was the greatest miMOI, and Both K EPEC and CI EPEC were 0.1 (Fig. 2b).

### Application of bacteriophage

The application of bacteriophage was used to assess the ability of bacteriophages in infecting the host bacteria on various food matrix. Food samples were artificially contaminated with EPEC, and bacteriophage used in this application was BL EPEC with MOI value of 0.01. The result showed that BL EPEC was able to reduce the bacteria concentration on lettuce and milk samples with reduction above 0.24log_10_. However, BL EPEC was not able to reduce bacteria concentration on tofu, chicken meat and shrimp samples (Table 1).

**Table 1:** The effectiveness of BL EPEC to various food samples in reducing EPEC

Samples	Control (CFU/mL)	Number of bacteria (CFU/mL)		Reduction (%)		Log reduction (log_10_)	
		Room temp.	4 °C	Room temp.	4 °C	Room temp.	4 °C
Lettuce	3.08 × 10^8^	1.76 × 10^8^	7.8 × 10^6^	42.86	74.68	0.24	0.60
Milk	2.08 × 10^8^	3 × 10^6^	1 × 10^6^	98.56	99.52	1.84	2.32
Tofu	TMTC	3 × 10^6^	TFTC	TFTC	TFTC	TFTC	TFTC
Chicken meat	2.85 × 10^8^	TFTC	TFTC	TFTC	TFTC	TFTC	TFTC
Shrimp	2.3 × 10^8^	TFTC	TFTC	TFTC	TFTC	TFTC	TFTC

*TMTC* too many to count; *TFTC* too few to count

## Discussions

In this study, bacteriophage titers were observed between 10^8^ and 10^9^ PFU/mL. This variation could be caused by differences in bacteriophage stability under certain conditions. Clear plaques were also formed on the agar plate, indicating the presence of lytic bacteriophages [14].

In general, most bacteriophages are stable under pH 4–11 [15]. BI EPEC, BL EPEC, and CS EPEC had a broader pH range compared to the other bacteriophages used in this study. The difference between bacteriophage stability in various pH can be caused by the difference in each isoelectric point. Bacteriophages were most likely to form aggregate when the pH was lesser or equal to their isoelectric point [16], hence decreasing their effectiveness to infect bacterial cells. As for thermal stability, all of bacteriophage samples showed a high tolerance toward high temperature. Denaturation of bacteriophage may occur as bacteriophage is mainly composed of nucleic acid and proteins. Upon showing a high tolerance towards high temperature, it was most likely that bacteriophages in this study were able to retain their native folded state when exposed to high temperature [17]. The stability of bacteriophages in this study was similar to previously reported phages having stability in pH 4–11, 60 °C [18] and pH 4–12, 70 °C [11].

Based on the TEM results, bacteriophages in this study most likely belonged to the *Caudovirales* order. Td is used to differentiate *Myoviridae* (td  ≥  16 nm) and *Siphoviridae* (td  <  16 nm) [19]. From this, it could be assumed that K EPEC bacteriophage belonged to the *Siphoviridae*, while BI EPEC and CS EPEC belonged to the *Myoviridae*. However, further analysis such as DNA sequencing is required to confirm the classification of each bacteriophages [20].

Stability of bacteriophages without the presence of a host was observed. Based on the results, BL EPEC bacteriophage exhibited the least reduction. In a study conducted by Huang et al. [11], titer of phage LPSE1 was reduced for 0.5 log_10_ after 168 h of incubation, with a reduction rate of 2.9  ×  10^–3^ log_10_ per hour.

The miMOI was estimated to be the lowest concentration of phage particles that can inhibit bacterial growth. Each tested phage showed lytic activity against EPEC Nmr-2, and all bacterial growth decreased as the MOI increased. The most effective miMOI was found from BL EPEC and CS EPEC, which was 0.01. Synnott et al. [21] and Bicalho et al. [12] also stated that miMOI value from *Staphylococcus aureus* and *Escherichia coli* phage was 0.01 and the most ideal MOI was the lowest. A greater MOI value will result in a rapid lytic activity as the bacteriophage replicates faster [22].

The effectiveness of application of bacteriophage as food preservative depended on several factors, such as food matrix, surface area, contact time, structure, bacterial load, dose of bacteriophage, and presence of other compounds [20, 23]. Based on the results, food samples lettuce and milk showed a compelling reduction of bacteria both at 4 °C and room temperature (Table 1). However, the reduction on other samples was below the detectable level. It might be happened due to the capability of phages in lysing the entire bacteria. Bacterial suspension that was added into food samples might be trapped within the protein network of the matrix, therefore the phages were not able to reach the bacteria [13, 24]. At a temperature  <  12 °C, phages were unable to infect host cells because the viable cells were inactivated, and phage requires the active growth phase of bacteria for replication [25, 26]. Phages that were kept at cold temperatures were more stable and still had a high titer compared to room temperature storage. However, the lytic activity at or above 15 °C is more effective in reducing the number of bacteria found in food because phage replication still occurs [23, 27]. Further research is required regarding the application of phage to improve food safety including experiments in different food matrices.

In conclusion, all of the bacteriophages were consider stable under specific pH, thermal and storage conditions with high titer. From five bacteriophages that had been purified and characterized, BL EPEC bacteriophage showed the greatest potency and had promising results to be used as a food preservative. BL EPEC bacteriophage showed high pH and thermal tolerance with the least reduction after 168 h of incubation without the presence of a bacterial cell host. Application of BL EPEC into various foods also effective to reduce bacterial growth.

## Limitation

Further study is still needed to be done to screen the effectiveness of this phage against other food-borne pathogens. Application of this phage in other variety of food also need to be explored. Molecular characterization also needs to be conducted for each bacteriophages.

## Data Availability

The datasets used and/or analysed during the current study are available from the corresponding author on reasonable request.

## Abbreviations

EPEC: Enteropathogenic *Escherichia coli*
LB: Luria–Bertani medium
miMOI: Minimum multiplicity of infection
SM: Sodium–Magnesium buffer
